# Necroptosis Contributes to Persistent Inflammation During Acute Leptospirosis

**DOI:** 10.3389/fimmu.2022.810834

**Published:** 2022-03-22

**Authors:** Suman Kundu, Advait Shetty, Maria Gomes-Solecki

**Affiliations:** ^1^ Department of Microbiology, Immunology and Biochemistry, The University of Tennessee Health Science Center, Memphis, TN, United States; ^2^ Department of Pharmaceutical Sciences, The University of Tennessee Health Science Center, Memphis, TN, United States

**Keywords:** Necroptosis, *L. interrogans*, Apoptosis, *L. biflexa*, Leptospirosis, Inflammation, MLKL, RIP3

## Abstract

Leptospirosis is an emerging infectious disease. Recently, canine and human leptospirosis outbreaks were reported in California and New York, respectively. In this study we evaluated the role that cell death processes play in the inflammatory response to *Leptospira*. Groups of male C3H/HeJ mice were infected with pathogenic *L. interrogans* and non-pathogenic *L. biflexa* for 24 and 72 hours; inflammatory processes were characterized for apoptosis and necroptosis by flowcytometry of spleen cells and were further assessed for expression of biomarkers of necroptosis by western blot. We found that pathogenic *L. interrogans* promotes apoptosis in myeloid neutrophils and monocytes at 24h and 72h post-infection, whereas *L. biflexa* promotes apoptosis of myeloid monocytes only at 24h post-infection. It is interesting that the immune cells undergoing the common programmed cell death pathway (apoptosis) are the cell types which were not increased in frequency in spleen of mice infected with *L. interrogans* (neutrophils) and *L. biflexa* (monocytes) in our previous study. The same trend was observed with pathogenic *L. interrogans* inducing necroptosis of myeloid neutrophils in addition to monocytes and macrophages at 24h and/or 72h post-infection, whereas *L. biflexa* promoted this pro-inflammatory cell death process in monocytes and macrophages only at 24h post-infection. Thus, early apoptosis and necroptosis of these cell types may explain its absence in frequency in spleen. Furthermore, at 24h and 72h, expression of the necroptosis molecular biomarkers p-MLKL, p-RIP1 and p-RIP3 was increased post infection with pathogenic *L. interrogans*. These data suggest that the underlying cell death processes involved in immune responses to pathogenic *Leptospira* contribute directly to persistent inflammation during the early stages of leptospirosis.

## Introduction

Leptospirosis, a neglected emerging disease with worldwide distribution that affects virtually all vertebrates is caused by pathogenic *Leptospira* spp and is associated with robust inflammation orchestrated by chemo-cytokine release, recruitment of different immune cells as well as cellular processes like phagocytosis and NET/MET formation (1, 2). Human leptospirosis ranges in severity from a mild, self-limited febrile illness to a fulminant life-threatening disease (3). The host response to infection is comprised of immune activation, inflammation and death of immune cells engaged in the process. Cell death often determines the outcome of pathogenesis (4–6). Although cell death is induced primarily as a defense mechanism and is often associated with pathogen removal (5), the type of cellular death (apoptosis, necroptosis, pyroptosis) can either be beneficial or detrimental to the host and it determines the extent of inflammation and disease progression.

The best studied programmed cell death is apoptosis which is regulated by different pro and anti-apoptotic factors like BAX, Bcl-2 and certain caspases like Caspase 3, 7 and 8 (7–9). In contrast, necroptosis involves a caspase independent pathway comprising major molecules such as Mixed Lineage Kinase Domain-Like (MLKL) protein and Receptor Interacting Serine/Threonine Kinase (e.g., RIP1 and RIP3) (9, 10). RIP1 kinase is also found to be engaged in apoptosis whereas RIP3 and MLKL solely contributes to necroptosis (11). One of the major foundations of necroptosis is the formation of the necroptotic complex between the RIP1-RIP3-MLKL; phosphorylation of the MLKL protein finally prompts membrane pore formation, cells undergo osmotic pressure changes with its environment and Danger Associated Molecular Pattern (DAMP) are released which further increases inflammation (12–14). Moreover, initial interaction between Pathogen Associated Molecular Pattern (PAMP like LPS) with Pattern recognition receptor (PRR such as TLRs, NLRs) determine the switch between shared or mutually regulated cell death processes (15, 16). In this context, cell death processes like apoptosis tend to lead to resolution of inflammation, whereas necroptosis lead to persistent inflammation thereby prolonging disease (5, 6, 10, 17). Thus, a new interesting concept emerged suggesting that even after their death, immune cells contribute to inflammation processes and benefit the host.

Our recent work supports that pathogenic *Leptospira* induce splenomegaly by recruiting large numbers of immune cells in this secondary lymphoid organ and activating the chemo-cytokine response (2). Understanding the fate of those immune cells in spleen can further lead to understanding persistent inflammation in the host during leptospirosis. Induction of apoptosis in macrophages and fibroblasts by pathogenic *Leptospira* was evident from earlier studies in mice and suggested that persistent infection can lead to cellular necrosis depending on virulence of the *Leptospira* strains (1, 18, 19). The goal of the current study was to analyze the cell death processes involved in splenic inflammation of C3H-HeJ mice infected with pathogenic and non-pathogenic *Leptospira* in the first three days of infection.

## Materials and Methods

### Animals

C3H-HeJ male mice were purchased from The Jackson Laboratory (Bar Harbor, ME). Experimental animals were maintained and used in a pathogen-free environment in compliance with the University of Tennessee Health Science Center Institutional Animal Care and Use Committee Protocol no. 19-0062.

### Bacterial Culture and Infections

Saprophytic *Leptospira biflexa* serovar Patoc (ATCC 23582) and pathogenic *Leptospira interrogans* serovar Copenhageni strain Fiocruz L1-130 (originally isolated from a patient in Brazil, passage 2 after hamster infection) was cultured according to a protocol described previously (20). *Leptospira* was enumerated by dark-field microscopy (Zeiss USA, Hawthorne, NY) using a Petroff- Hausser counting chamber. 10-week-old male mice were inoculated intraperitoneally (IP) with a ~ 10^8^ dose of bacteria resuspended in PBS); controls received sterile PBS, pH7.5.

### Flow Cytometry

Spleens were collected from different groups of mice after euthanasia and processed immediately for flow cytometry staining. Briefly, spleens were chopped and teared with frosted slides to prepare single cell suspensions and RBC lysis was performed as described previously (2). Live/dead cell stain was performed, and cells were counted using a Luna counter (Logos Biosystems, South Korea). Approximately 10^6^ cells/well were seeded in a 96 well microtiter plate for flow cytometry staining. Blocking was performed with anti-mouse CD16/32 antibody (1:50) and incubated for 15-20 min on ice in staining buffer. Surface staining was performed against different cell surface markers using specific primary conjugated antibody, the compensation beads were used to stain specific fluorochrome conjugated antibodies and incubated in the dark for 30 minutes at 4°C. Equal numbers of live and dead (1:1) cells were stained with Annexin V/PI for compensation. Cells were washed twice with FACS staining buffer after incubation. Cells were resuspended in annexin binding buffer and stained with Annexin V (1:50) and Propidium Iodide (PI, 1:200) for 15 minutes at room temperature in a dark environment. Stained cells were acquired in a BioRad ZE5 Cell analyzer and data were analyzed using Flow Jo software. The fluorochrome conjugated antibody panel (**Table S1**) and the gating strategy (**Figure S1**) used are presented in the supplementary section. One representative plot for cell death is provided in the **Figure S1** where quadrant 3 (Annexin V+PI-) represents the zone of apoptosis and quadrant 2 (Annexin V+PI+) represents the zone of secondary apoptosis or necroptosis (14).

### Western Blot

Whole spleen cells were pelleted by centrifugation and the supernatant was discarded. The pellet was washed twice with PBS and was dissolved in Cell lysis buffer (Cell Signaling Technology, USA) supplemented with protease inhibitor cocktail (1X) and 1mM PMSF (Thermo Fisher Scientific, USA). The lysed cell suspension was kept on ice for 30 minutes and cellular debris were removed by centrifugation at 10,000 rpm for 10-15 minutes. The protein concentration of the cell lysate was measured with DC protein assay kit (BioRad Laboratories, USA). Cell lysate of equal concentration and volume were mixed with 1X SDS loading buffer and boiled for 5 minutes at 95°C; samples were separated by 10% SDS-PAGE and transferred to PVDF. The membrane was probed with appropriate primary and HRP-conjugated secondary antibodies (**Table S2**), and developed with a chemiluminescent substrate solution (MilliporeSigma, USA). The signal was captured in a Chemidoc Image Analyzer (BioRad Laboratories, USA). The same membrane was re-probed 3-5 times after stripping the membrane (Thermo Fisher Scientific, USA). Densitometric analysis was performed for different proteins of interest using ImageJ software (NIH, USA) and representative histograms were generated using β-Actin as control.

### STRING Protein-Protein Interaction Network Analysis

A total of 17 query proteins out of which 14 were increased chemo-cytokines in serum from our previous study (2) and 3 are elevated key necroptotic proteins from the present study at 72 h post infection with *L. interrogans* were used to generate a STRING proteome map using the online database (string-db.org). In these networks, the nodes are the proteins and the edges indicate the interactions between proteins as they were present in STRING (level of confidence >0.400). It contains information about known and predicted, direct physical, and indirect functional protein-protein interactions which are represented in different colored lines.

### Statistical Analysis

Statistical analysis was performed by GraphPad Prism software using unpaired student t-test with Welch’s correction between experimental groups. Significance was analyzed between infected versus uninfected groups and between the *L. biflexa* and *L. interrogans* infected groups where p value <0.05 is considered significant.

## Results

### Pathogenic *L. interrogans* Promotes Apoptosis in Myeloid Neutrophils and Monocytes at 24h and 72h Post-Infection, Whereas *L. biflexa* Promotes Apoptosis of Myeloid Monocytes Only at 24h Post-Infection

**Figure 1:**
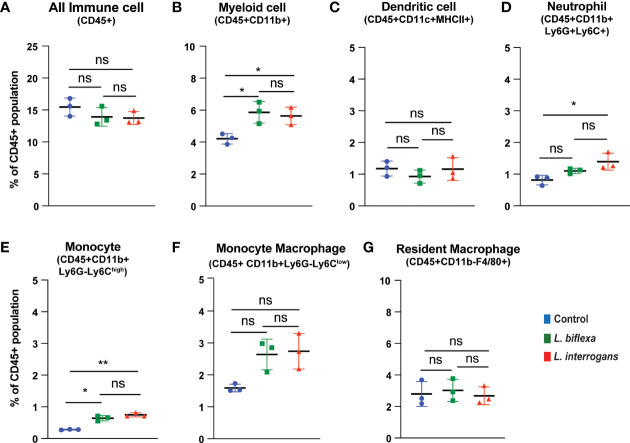
Apoptotic cell death among different immune cells in spleen at 24h post infection. Annexin V^+^/Propidium Iodide (PI)^-^ cells were considered as zone of apoptosis in **(A)** All Immune Cells, **(B)** Myeloid cell, **(C)** Dendritic cell, **(D)** Neutrophil, **(E)** Monocyte, **(F)** Monocyte-Macrophage and **(G)** Resident Macrophage. Plots are represented as % of CD45+ population. Statistical analysis was performed using unpaired student t-test with Welch’s correction, *p < 0.05, **p < 0.01; n = 3 mice per group. ns, not-significant. Data represents one of two independent experiments; N= 7 mice per group.

**Figure 2:**
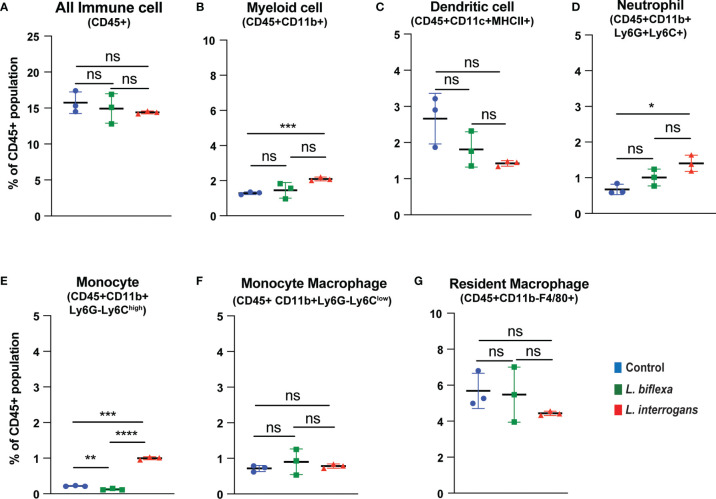
Apoptotic cell death among different immune cells in spleen at 72h post infection. Annexin V^+^/PI^-^ cells were considered as zone of apoptosis in **(A)** All Immune Cells, **(B)** Myeloid cell, **(C)** Dendritic cell, **(D)** Neutrophil, **(E)** Monocyte, **(F)** Monocyte-Macrophage and **(G)** Resident Macrophage. Plots are represented as % of CD45+ population. Statistical analysis was performed using unpaired student t-test with Welch’s correction, *p < 0.05, **p < 0.01, ***p < 0.001, ****p < 0.0001; ns, not-significant; n = 3 mice per group. Data represents one of two independent experiments; N = 7 mice per group.

Mice (10 week old, n=7 per group) were infected with a sublethal dose of bacteria intraperitoneally, spleen was excised and the cell death process was assessed by flowcytometry. Annexin V positive cells (Annexin V^+^/PI^-^) were marked as zone of apoptosis and was measured at 24h (**Figure 1**) and 72h (**Figure 2**) post-infection. At 24h and 72h post-infection, apoptosis was significantly increased in splenic myeloid cells (**Figures 1B**, **2B**), neutrophils (**Figures 1D**, **2D**) and monocytes (**Figures 1E**, **2E**) from mice infected with *L. interrogans* compared to uninfected controls. Increased apoptosis was also observed in splenic myeloid cells (**Figure 1B**) and monocytes (**Figure 1E**) of mice inoculated with *L. biflexa* at 24h post infection, whereas at 72h post infection, monocyte apoptosis was significantly decreased (**Figure 2E**). No differences in apoptosis were observed in dendritic cells, monocyte macrophages and resident macrophages at 24h or 72h post infection with either *Leptospira* species.

### Pathogenic *L. interrogans* Promotes Necroptosis in Myeloid Neutrophils, Monocytes, and Macrophages at 24h and/or 72h Post-Infection, Whereas *L. biflexa* Promotes Necroptosis in Monocytes and Macrophages Only at 24h Post-Infection

**Figure 3:**
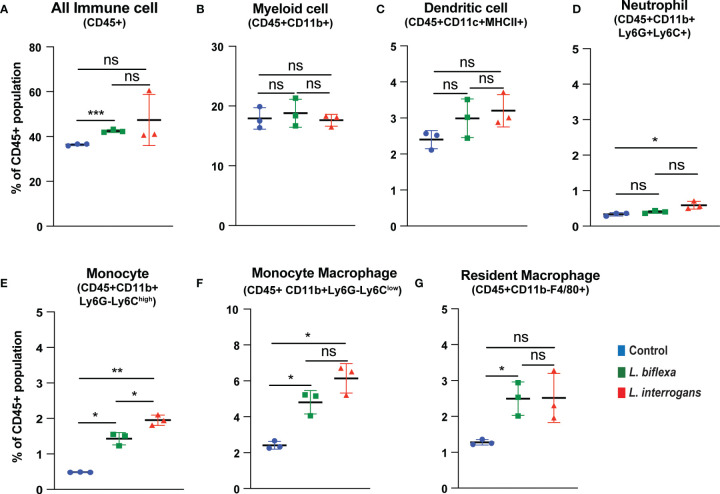
Necroptotic cell death among different immune cells in spleen at 24h post infection. Annexin V^+^/PI^+^ cells were considered as zone of necroptosis in **(A)** All Immune Cells, **(B)** Myeloid cell, **(C)** Dendritic cell, **(D)** Neutrophil, **(E)** Monocyte, **(F)** Monocyte-Macrophage and **(G)** Resident Macrophage. Plots are represented as % of CD45+ population. Statistical analysis was performed using unpaired student t-test with Welch’s correction, *p < 0.05, **p < 0.01, ***p < 0.001; n = 3 mice per group. ns, not-significant. Data represents one of two independent experiments; N = 7 mice per group.

**Figure 4:**
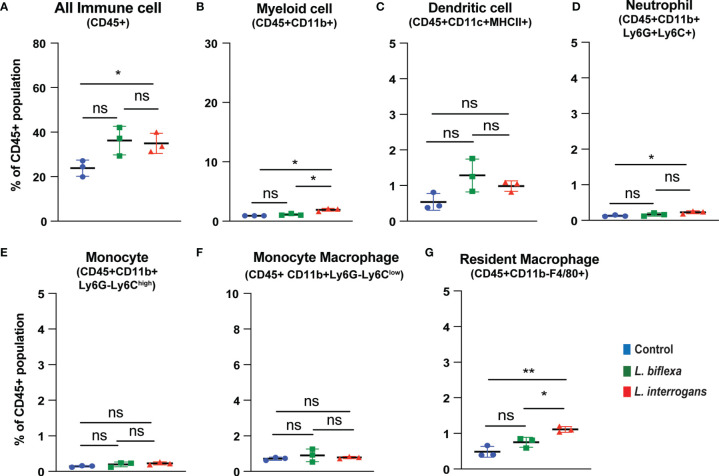
Necroptotic cell death among different immune cells in spleen at 72h post infection. Annexin V^+^/PI^+^ cells were considered as zone of necroptosis in **(A)** All Immune Cells, **(B)** Myeloid cell, **(C)** Dendritic cell, **(D)** Neutrophil, **(E)** Monocyte, **(F)** Monocyte-Macrophage and **(G)** Resident Macrophage. Plots are represented as % of CD45+ population. Statistical analysis was performed using unpaired student t-test with Welch’s correction, *p < 0.05, **p < 0.01; n = 3 mice per group. Data represents one of two independent experiments; N= 7 mice per group. ns, not-significant.

Mice (10 week old, n=7 per group) were infected with a sublethal dose of bacteria intraperitoneally, spleen was excised and the cell death process was assessed by flowcytometry. Splenocytes from infected mice were analyzed for necroptosis (Annexin V^+^/PI^+^) at 24h (**Figure 3**) and 72h (**Figure 4**) post-infection by measuring the Annexin V and Propidium iodide (PI) double positive quadrant by flowcytometry. For *L. interrogans* infected mice, necroptosis was significantly increased in splenic neutrophils (**Figure 3D**), monocytes (**Figure 3E**) and monocyte-macrophages (**Figure 3F**) at 24h post-infection compared to uninfected controls, whereas at 72h it was significantly increased in overall immune cells (**Figure 4A**), myeloid cells (**Figure 4B**), neutrophils (**Figure 4D**) and resident macrophages (**Figure 4G**). For mice inoculated with *L. biflexa*, a significant increase in necroptosis was observed only at 24h in overall splenic immune cells (**Figure 3A**), monocytes (**Figure 3E**), monocyte-macrophages (**Figure 3F**) and resident macrophages (**Figure 3G**). No differences in necroptosis were observed in dendritic cells at 24h or 72h post infection with either *Leptospira* species. The flowcytometric analysis of overall apoptosis (Annexin V^+^/PI^-^) and necroptosis (Annexin V^+^/PI^+^) in different immune cell types at 24h and 72h post infection is summarized in **Table 1**.

**Table 1:** Summary of cell death processes in different immune cells after *Leptospira* infection.

	Apoptosis (Annexin V^+^/PI^-^)				Necroptosis (Annexin V^+^/PI^+^)			
	*L. biflexa*		*L. interrogans*		*L. biflexa*		*L. interrogans*	
**Phenotype**	24h	72h	24h	72h	24h	72h	24h	72h
All Immune Cells (CD45+)	ns	ns	ns	ns	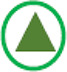	ns	ns	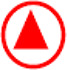
Myeloid cell (CD45+CD11b+)	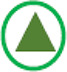	ns	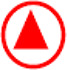	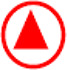	ns	ns	ns	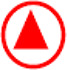
Dendritic cell (CD45+ CD11c+MHCII+)	ns	ns	ns	ns	ns	ns	ns	ns
Neutrophil (CD45+CD11b+Ly6G+Ly6C+)	ns	ns	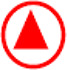	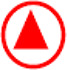	ns	ns	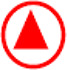	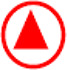
Monocyte (CD45+CD11b+Ly6G-Ly6C^high^)	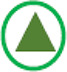	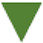	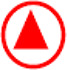	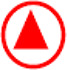	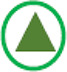	ns	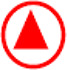	ns
Monocyte-Macrophage (CD45+CD11b+Ly6G-Ly6C^low^)	ns	ns	ns	ns	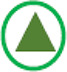	ns	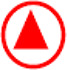	ns
Resident Macrophage (CD45+CD11b-F4/80+)	ns	ns	ns	ns	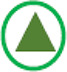	ns	ns	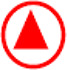

L. biflexa (green arrowhead) and L. interrogans (red arrowhead) infection in comparison to uninfected control at 24h and 72h; PI, represents Propidium Iodide; a circle around the arrowhead highlights increases; ns, represents non-significance.

### Expression of Major Necroptosis Biomarkers in Splenocytes Is Much Higher at 24h and 72h Post Infection With *L. interrogans* Than *L. biflexa*

**Figure 5:**
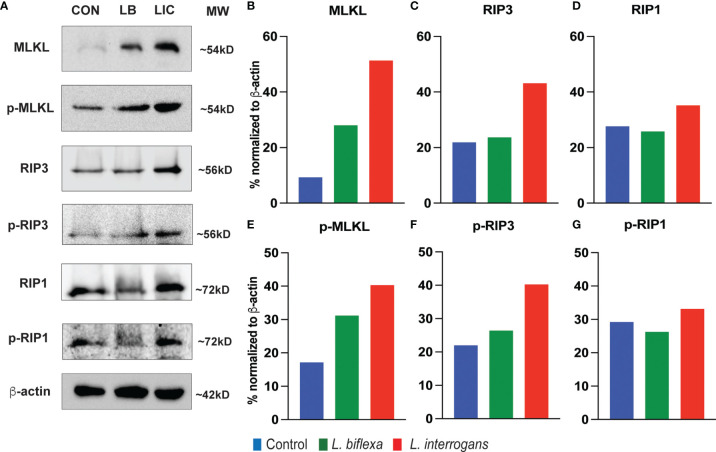
Protein expression of key biomarkers of necroptosis at 24h post infection. **(A)** represents immunoblots of protein lysate (whole spleen cells) from uninfected Control (CON), nonpathogenic *L. biflexa* (LB) and pathogenic *L. interrogans* (LIC) infected groups. **(B–G)** represents the densitometric histograms normalized to β-actin expression control using ImageJ software. Legend: MLKL (Mixed Lineage Kinase Domain-Like protein), Receptor Interacting Serine/Threonine Kinase (RIP1 and RIP3); p, phosphorylated.

**Figure 6:**
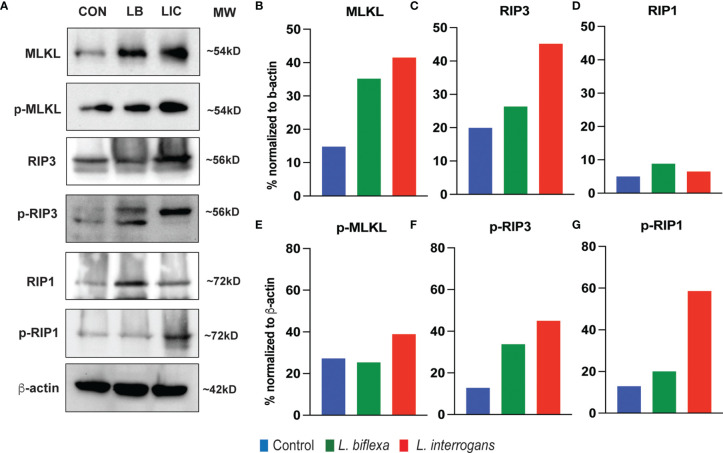
Protein expression of key biomarkers of necroptosis at 72h post infection. **(A)** represents the immunoblots of protein lysate (whole spleen cells) from uninfected Control (CON), nonpathogenic *L. biflexa* (LB) and pathogenic *L. interrogans* (LIC) infected groups. **(B–G)** represents the densitometric histograms normalized to β-actin expression control using ImageJ software. Legend: MLKL, Mixed Lineage Kinase Domain-Like protein; Receptor Interacting Serine/Threonine Kinase (RIP1 and RIP3), p, phosphorylated; MW, molecular weight; kD, kilodalton.

Major necroptosis marker proteins (MLKL, RIP1 and RIP3) and their phosphorylated forms were analyzed in whole spleen cell lysate from mice infected with *L. interrogans* and *L. biflexa* by western blot at 24h and 72h post infection, in comparison with uninfected control (**Figures 5**, **6**). The phosphorylated biomarkers assemble the necroptotic complex that form the membrane pore that releases DAMPs. At 24h post-infection, we found that the total protein against all three necroptosis biomarkers (MLKL, RIP3 and RIP1) and their phosphorylated forms (p-MLKL, p-RIP1 and p-RIP3) were increased in *L. interrogans* infected splenocyte lysates (**Figures 5A–G**), whereas only MLKL and p-MLKL was increased in *L. biflexa* infected lysates (**Figures 5B, E**). At 72h post-infection, we found that the total protein of two major necroptosis biomarkers (MLKL and RIP3) and all three phosphorylated forms of necroptosis biomarkers (p-MLKL, p-RIP1 and p-RIP3) were elevated in *L. interrogans* infected splenocyte lysates (**Figures 6A–G**), though p-RIP3 and p-RIP1 were slightly increased in *L. biflexa* infected lysates, but p-MLKL was not increased (**Figures 6E–G**). **Table 2** represents the overall summary of protein expression of key necroptotic molecules in C3H/HeJ mice spleen at 24h and 72h post infection with *L. interrogans* and *L. biflexa* compared to control.

**Table 2:** Summary of protein expression of key necroptotic molecules during *Leptospira* infection.

Key Necroptosis Markers	Protein Expression			
	*L. biflexa*		*L. interrogans*	
	24h	72h	24h	72h
MLKL	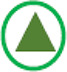	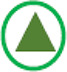	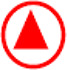	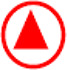
p-MLKL	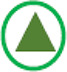	**≈**	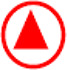	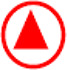
RIP3	**≈**	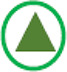	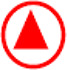	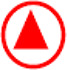
p-RIP3	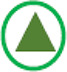	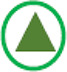	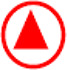	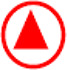
RIP1	**≈**	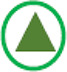	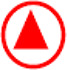	**≈**
p-RIP1	**≈**	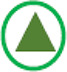	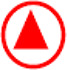	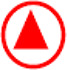

% Normalized to β-actin ± 3 is considered as equivalent (**≈**) between infected and uninfected; comparison between L. biflexa (green arrowhead) and L. interrogans (red arrowhead) compared to control at 24h and 72h post-infection; a circle around the arrowhead highlights increases.

### Modeling of Protein-Protein Networking Between Major Chemo-Cytokines and Necroptotic Molecules 72h Post Infection With Pathogenic *L. interrogans*

**Figure 7:**
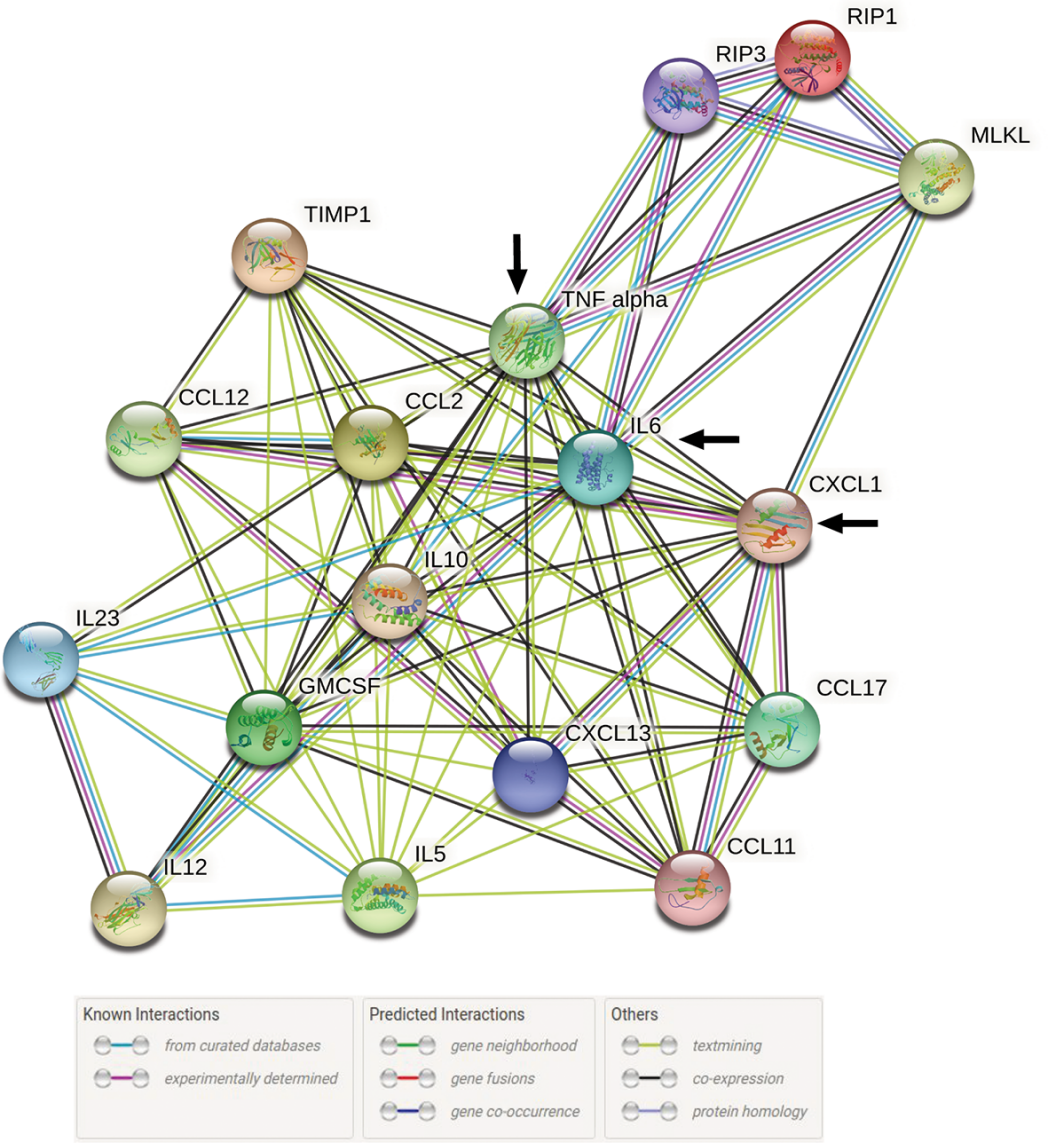
STRING proteome analysis of chemo-cytokines with major necroptosis molecules at 72h post infection. A total of 17 proteins (14 elevated chemokines and cytokines in serum of infected mice (2) and 3 elevated necroptotic proteins in spleen) were analyzed by protein network analysis using STRING online database (string-db.org) tools. Representative protein-protein network model is representative of total number of Nodes = 17 and Edges = 84 with medium level of confidence (0.400). Black arrow indicates the direct interaction between IL-6, TNF-α and CXCL1/KC with necroptotic molecules MLKL, RIP1 and RIP3.

We evaluated the interactions of increased chemo-cytokines present in serum 72h post infection with *L. interrogans* from our previous study (2) with the major necroptotic markers MLKL, RIP3 and RIP1 elevated in the spleen at 72h post infection in the present study through STRING proteome analysis (**Figure 7**). This analysis informs on known and predicted protein-protein interactions as well as direct physical and indirect functional interactions. Cytokines TNF-α and IL-6 from the previous study (2) showed strong interactions (experimentally determined interactions represented by the purple line in **Figure 7** with all 3 key necroptotic molecule MLKL, RIP3 and RIP1 (present study). Furthermore, CXCL1/KC directly established a strong association with necroptotic molecule MLKL. The network map also represents an indirect association between other chemo-cytokines with the necroptotic proteins associated with *L. interrogans* infection.

## Discussion

The intricate interactions between host and pathogen depends on several immune factors and signaling intermediates which can either be beneficial to the host if the response succeeds in eliminating the pathogen or detrimental, if the pathogen evades immune eradication, proliferates and boosts the host patho-inflammatory response (4, 5, 15). The goal of this study was to understand whether cell death processes engaged during the onset of Leptospirosis are apoptotic inflammation-neutral or necroptotic pro-inflammatory. In our previous study we observed gross anatomic increases in spleen (splenomegaly) at 24h and/or 72h post-infection with pathogenic *Leptospira*, in contrast to infection with a saprophytic species (2). In this study we evaluated the immune cell phenotypes undergoing different cell death processes in spleen of mice at 24h and 72h post-infection with *L. interrogans* in comparison with *L. biflexa* and non-infected controls. We used male mice in our studies as they are more susceptible to Leptospirosis as observed previously in male hamsters (21).

Previously we observed recruitment to spleen of all myeloid cells except neutrophils at 24h and 72h post-infection with *L. interrogans*, in contrast to infection with *L. biflexa* which led to increased populations of myeloid cells at 24h or 72h except monocytes and monocyte-macrophages (2). In this study we found that pathogenic *L. interrogans* promotes apoptosis in myeloid neutrophils and monocytes at 24h and 72h post-infection, whereas *L. biflexa* promotes apoptosis of myeloid monocytes only at 24h post-infection. It is interesting that the immune cells undergoing the common programmed cell death pathway (apoptosis) are the cell types which were not increased in frequency in spleen of mice infected with *L. interrogans* (neutrophils) and *L. biflexa* (monocytes). When we looked at the pro-inflammatory cell death process, we observed the same trend with pathogenic *L. interrogans* inducing necroptosis of myeloid neutrophils in addition to monocytes and macrophages at 24h and/or 72h post-infection, whereas *L. biflexa* promoted necroptosis in monocytes and macrophages only at 24h post-infection. Thus, early apoptosis and necroptosis of these cell types may explain its absence in frequency in this secondary lymphoid organ. Previous reports suggest that pathogenic *L. interrogans* infection induced apoptosis in the macrophage and mouse tissues (18, 22–25) and both *L. interrogans* and saprophytic *L. biflexa* triggers delayed apoptosis in neutrophils by releasing proinflammatory cytokines (26). Moreover, data suggests that in addition to apoptosis, *L. interrogans* but not *L. biflexa* infection, led to necrosis in fibroblasts, macrophages and peripheral blood mononuclear cells of murine and human origin (19, 24, 27). It is known that neutrophils play an important role in trapping extracellular *L. interrogans* early in infection (28). Furthermore, *L. interrogans* was observed on the surface of neutrophils and not phagocytized whereas *L. biflexa* was phagocytized (26) which further shows the importance of these cells in clearance of *Leptospira*. Here we show that apoptosis and necroptosis of neutrophils only in *L. interrogans* infected mice suggests that early removal of these cells from the innate immune cell repertoire and the enhanced inflammation promoted by necroptosis (29) may contribute to *L. interrogans* evasion, dissemination, pathogenesis and activation of the adaptive immune system characteristic of acute leptospirosis. We speculate that necroptosis may play a role in phagocytosis impairment by neutrophils in the presence of *L. interrogans*. The chemo-cytokines elevated at 72h post infection with pathogenic *L. interrogans* (2) are associated with the elevated major molecules of necroptosis described in the current study (**Figure 7**) which indicates an intricate exchange of inflammatory signatures in the regulation of inflammatory cell death process involved during persistent inflammation. Therefore, persistent inflammation (splenomegaly) during leptospirosis is a result of a large chemo-cytokine release in the system and necroptosis activation (induction of MLKL/RIP3) which can further lead to membrane pore formation leading to cell death (30, 31).

While flowcytometry of the cellular repertoire distinguished between different cell death processes among diverse immune cell types, proper activation of the necroptosis process was further dissected by western blot analysis of whole spleen cell lysates after *Leptospira* infection. In both 24h and 72h, expression of all the molecular biomarkers of necroptosis like MLKL, RIP1 and RIP3 was increased post infection with pathogenic *L. interrogans*. The increased expression of the active phosphorylated forms p-MLKL, p-RIP1 and p-RIP3 after pathogenic infection activates the necroptotic complex necessary to initiate the membrane pore formation in those immune cells to release inflammatory arsenals (DAMPs) necessary to maintain an inflammatory milieu and thereby invite the adaptive response which results in aggravated pathogenicity (12). These biomarkers were also increased in spleen cell lysates stimulated with other pathogens (5, 17, 29, 32). The spotty increased expression of those key biomarkers at 24h and 72h post infection with the nonpathogenic *L. biflexa* suggests a discontinuous inflammatory process as the innate response is enough to provide protection. The key molecules involved in the process of necroptosis during acute Leptospiral infection could be a potential target for the development of future therapeutics.

## Data Availability Statement

The original contributions presented in the study are included in the article/**Supplementary Material**. Further inquiries can be directed to the corresponding author.

## Ethics Statement

Experimental animals were maintained and used in a pathogen-free environment in compliance with the University of Tennessee Health Science Center Institutional Animal Care and Use Committee Protocol no. 19-0062.

## Author Contributions

Conceptualization: SK. Experimental Investigation: SK and AS. Data Analysis: SK and MGS. Writing Original Draft and Editing: SK and MGS. Supervision and Funding Acquisition: MGS. All authors contributed to the article and approved the submitted version.

## Funding

This work was supported by the Public Health Service awards AI139267 (MGS), AI142129 (MG-S) and AI155211 (MGS), from the National Institute of Allergy and Infectious Diseases (NIAID) of the National Institutes of Health (NIH) of the United States of America.

## Author Disclaimer

The content of this manuscript is solely the responsibility of the authors and does not necessarily represent the official views of NIAID or NIH.

## Conflict of Interest

The following authors declare potential conflicts of interest: MGS (grants from federal agencies and employment).

The remaining authors declare that the research was conducted in the absence of any commercial or financial relationships that could be construed as a potential conflict of interest.

## Publisher’s Note

All claims expressed in this article are solely those of the authors and do not necessarily represent those of their affiliated organizations, or those of the publisher, the editors and the reviewers. Any product that may be evaluated in this article, or claim that may be made by its manufacturer, is not guaranteed or endorsed by the publisher.
