# Supplementary material for: Necroptosis Contributes to Persistent Inflammation During Acute Leptospirosis
Source: Front Immunol. 2022 Mar 22;13:810834. doi: 10.3389/fimmu.2022.810834 (PMC8980737; doi:10.3389/fimmu.2022.810834)
Supplement: Supplementary file 1 [file DataSheet_1.docx]

Supplementary Materials:


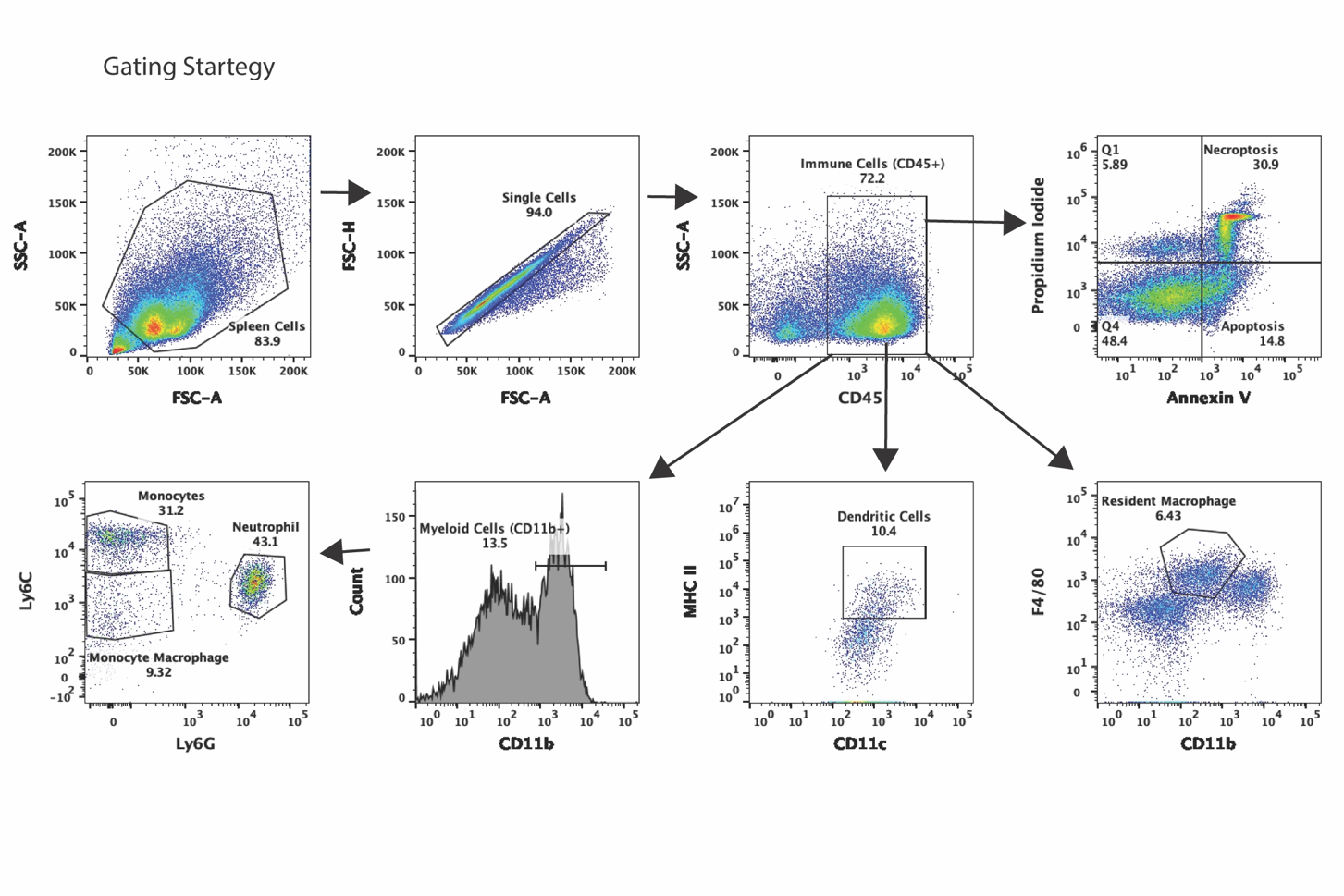


**Figure S1:** Gating strategy applied to analyze different cell death processes. Black arrow indicates the subsequent gating path for each immune cell types analyzed in this study. One representative figure (Upper extreme right) indicates the gated zone for Apoptosis (Annexin V+PI-) and Necroptosis (Annexin V+PI+) respectively from all immune cells. The Q4 (Quadrant 4) represents the live immune cells.

Table S1: List of fluorochrome conjugated antibodies and dyes

| **Marker** | **Fluorophore** | **Company** | **Catalog Number** |
| --- | --- | --- | --- |
| CD45 | Brilliant Violet 605 | Biolegend | 103140 |
| F4/80 | Brilliant Violet 785 | Biolegend | 123141 |
| CD11b | Brilliant Violet 711 | Biolegend | 101241 |
| CD11c | APC-Cy7 | TONBO biosciences | 25-0114-U100 |
| Ly6C | Brilliant Violet 510 | Biolegend | 128033 |
| Ly6G | PE | Biolegend | 127608 |
| MHC-II | PE-Cy7 | Biolegend | 107629 |
| Annexin V | FITC | Biolegend | 640906 |
| Propidium Iodide (PI) | - | Biolegend | 421301 |

Table S2: List of antibodies used for Western Blot

| Marker protein name | Dilution | Company | Catalog Number |
| --- | --- | --- | --- |
| MLKL | 1:2000 | Cell Signaling  Technology |  |
| phospho-MLKL | 1:1000 |  |  |
| RIP3 | 1:2000 |  | 47928T |
| phospho-RIP3 | 1:1000 |  |  |
| RIP1 | 1:1000 |  |  |
| phospho-RIP1 | 1:1000 |  |  |
| Secondary anti Rabbit IgG | 1: 3000 |  |  |
| β-Actin | 1:4000 |  | 4970S |
